# The Association Between Alzheimer's Disease and Epilepsy: A Narrative Review

**DOI:** 10.7759/cureus.30195

**Published:** 2022-10-11

**Authors:** Manisha Purushotham, Fatema Tashrifwala, Rahul Jena, Sunil Akshara Vudugula, Rutuja S Patil, Aditi Agrawal

**Affiliations:** 1 Internal Medicine, Kasturba Medical College, Manipal, IND; 2 Medicine, University of Mauritius, Belle Rieve, MUS; 3 Medicine, Bharati Vidyapeeth Medical College/Bharati Hospital, Pune, IND; 4 Internal Medicine, J.S.S. Medical College, Mysore, IND; 5 Medicine, Bharati Vidyapeeth Medical College Pune, Pune, IND; 6 Psychiatry, Boston University School of Medicine, Boston, USA

**Keywords:** levetiracetam, gaba, neurodegenerative disease, beta amyloid, tau protein, seizures, alzheimer's disease and epilepsy

## Abstract

Several studies have established the two-way relationship between the sporadic and familial forms of Alzheimer’s disease (AD) and epilepsy. However, a more robust connection exists between epilepsy and early onset familial AD (EOFAD). Still, the mechanisms underlying the same are not yet fully understood. Aging is also known to be associated with both AD and seizures. Seizures of any type can occur at any stage of AD and are six to 10 times more likely in patients with AD than in controls of a similar age group. Seizures can quicken cognitive decline and increase mortality, amplifying the medical and economic burden. It is, therefore, clinically essential to recognize and treat seizures early in these patients. However, diagnosis of seizures in AD is complicated by the difficulty in identifying non-motor focal seizures in patients with cognitive decline, problems with obtaining histories, low sensitivity of standard scalp electroencephalogram (EEG) methods, nonspecific cerebrospinal fluid (CSF) and radiological findings. This article has reviewed and summarised the existing literature on the association between AD and epilepsy pertaining to epidemiology, pathophysiological links, risk factors, modalities for diagnosis, and treatment strategies.

## Introduction and background

Alzheimer’s disease (AD) is a progressive neurodegenerative disease typified by loss of memory, language, behavior, and personality [1]. Globally, around 55 million people have dementia, with AD contributing to about 60-70% of the total. As the percentage of older people in the population is on the rise in nearly every nation, this number is expected to grow to 78 million in 2030 and 139 million in 2050 [2]. It is important to note that dementia is now the seventh leading cause of mortality worldwide and the one with the highest economic burden [3]. It is now increasingly apparent that undiagnosed and unprovoked seizures exist in a fair number of patients with AD and are eight to 10 times greater in patients with AD compared to the general population [4-7]. Reciprocally, those diagnosed with epilepsy have a 1.6 times higher risk than those without epilepsy of incident AD [8]. A study on the rat hippocampus has shown that epilepsy can increase the production of A-beta 40 amyloid protein [9]. Some studies suggest seizures can cause a decline in cognition in patients with AD [5]. This is supported by the Presentation of Epileptic Seizures in Dementia (PrESIDe) study, in which initially, patients with and without a suspicion of epilepsy performed similarly on cognitive tests. Still, at a 12-month follow-up, the patients suspected of epilepsy performed much worse on cognitive tests. The rates of unprovoked seizures were significantly elevated in younger individuals with the autosomal dominant form, in African Americans, in severe forms of the disease, and individuals with focal epileptiform findings on electroencephalogram (EEG) [10]. Moreover, subclinical epileptiform activity (epileptiform activity without seizures) predominantly localized to the temporal lobe was found in about 42% of the patients with AD that received extended neurophysiological monitoring [5]. It is worth noting that these subsets of patients also had an accelerated decline in cognition and executive function [5]. Therefore, screening patients for epileptiform activity is recommended to improve diagnosis and outcomes as only 10-22% of patients with AD have clinically detectable seizures [11]. This is in accordance with another study that showed most seizures in AD to be non-motor and, therefore, easily missed [12]. The true prevalence of it is difficult to estimate as fluctuation in cognition could be the only presentation of seizures which is more difficult to decipher in the AD setting, which independently causes cognitive dysfunction. The importance of identifying seizures in patients with AD is of therapeutic significance as anti-epileptic medication has shown to improve memory functions in patients with mild cognitive impairment (MCI) [13]. The focus of this review is to understand the association between AD and epilepsy, briefly explore the pathophysiological and molecular mechanisms underlying it as these are vital in developing treatment strategies, discuss various diagnosing modalities and summarise the current treatment strategies for epilepsy in patients with AD.

## Review

Pathophysiology

**Figure 1:**
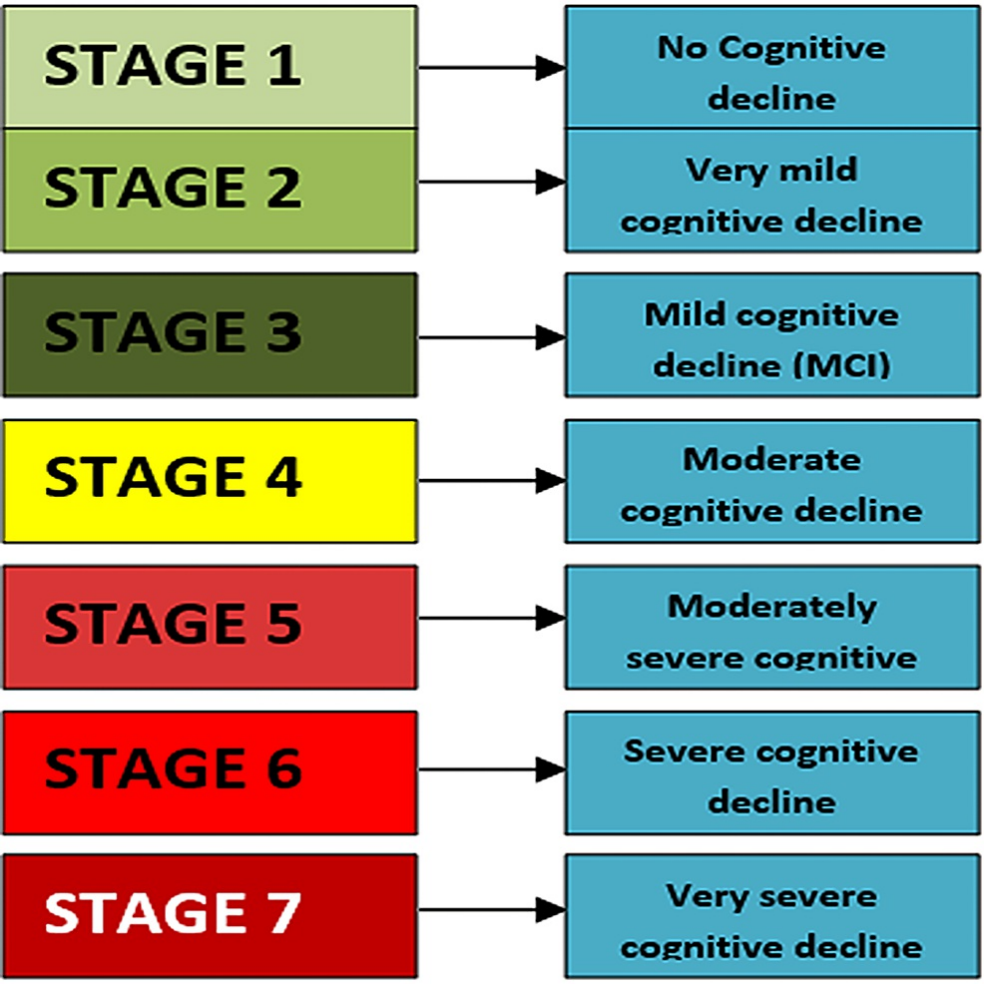
Global Deterioration Scale (Reisberg) Figure by Manisha Purushotham

The Global Deterioration Scale developed by Dr Barry Reisberg has seven stages and is depicted in Figure 1. The criteria for epilepsy as per the International league against epilepsy is seen summarized in Table 1.

**Table 1:** Criteria for Epilepsy

CRITERIA FOR EPILEPSY (International league against epilepsy)
At least two unprovoked (or reflex) seizures occurring more than 24 hours apart.
One unprovoked (or reflex) seizure and a probability of further seizures similar to the general recurrence risk (at least 60%) after two unprovoked seizures, occurring over the next ten years.
Diagnosis of an epilepsy syndrome

The pathophysiology linking AD and epilepsy is complex and not fully understood. The following factors have been described in the literature:

Beta-Amyloid

Twenty-five percent of people who develop epilepsy in late adulthood have no known cause and are diagnosed with late-onset epilepsy of unknown etiology (LOEU) [14]. People with LOEU have been shown to have amyloid pathology in the brain, with amyloid-β (Aβ) deposition increasing their risk of developing cognitive decline over the decades [14]. Histopathological hallmarks of AD are intracellular neurofibrillary tangles (NFTs) and extracellular formation of senile plaques composed of the Aβ peptide. Radioactive metal ions, for example copper, can catalyze the production of reactive oxygen species (ROS) when bound to Aβ [15]. The ROS thus produced, in particular the hydroxyl radical, is the most reactive and may contribute to oxidative damage on both the Aβ peptide and the surrounding molecules (proteins, lipids, neurons) [15]. Aβ is pro-epileptogenic at the oligomer stage, well before plaque deposition, and its accumulation fosters network hyperexcitability [14]. While low concentrations of Aβ facilitate the synaptic transmission, higher concentrations reduce synaptic activity. It is likely that in AD, a steady and small increase of Aβ prompts neuronal hyperexcitability. At higher levels of Aβ, synaptic dysfunction and inhibition occur, which becomes clinically apparent as cognitive impairment [16]. Epileptic discharges and seizures during prodromal stages of AD can be set off by Aβ oligomers and increase dynamically with Aβ deposition, supporting the speculation that Aβ-related epileptogenesis sets the stage for resulting neurodegeneration [14]. These discoveries appear to convert into clinical experience, with seizures happening now and again in patients with prodromal AD [14,16]. Thus Aβ is at the interface of epileptogenesis and neuronal loss. Aβ also has epileptogenic potential in the early stages of the amyloid cascade. Preclinical studies have demonstrated that Aβ oligomers can induce spontaneous epileptiform discharges and clinically overt seizures. The epileptogenic potential of oligomers can depend on oligomerization status, threshold concentrations, and interaction with other proteins [17,18]. Production of Aβ species is activity-dependent and consistently increases with neuronal firing [18]. Epileptiform activity favors plaque deposition and Aβ plaque deposition, in turn, alters neuronal signaling, maintains a dynamic environment in equilibrium with oligomers, and promotes epileptiform activity, creating a vicious cycle. The contribution of every single item to the vicious cycle is yet to be fully understood, given that experimental models do not satisfactorily replicate mild Alzheimer's disease stages. Dissection of each mechanism might aid the development of multitarget strategies [14]. Glycogen synthase kinase-3 (GSK-3) is a proline-rich serine/threonine kinase and is essential in the pathogenesis of AD as it acts as a bridge between amyloid and tau; amyloid activates GSK 3, which in turn phosphorylates tau [19]. Many studies have already suggested that Tau and Amyloid play a role in epileptogenesis, and it can be deduced that GSK 3 has a role in developing unprovoked seizures in AD [19]. mTOR A serine/threonine kinase, expressed in multiple cell types, generates Aβ42, and its hyperactivation is reported in both temporal lobe epilepsy (TLE) and AD [20]. 

Tau Protein

Tau protein is located in axons, a microtubule-associated protein type II; the MAPT gene encodes it on chromosome 17q21 [16]. Physiologically Tau protein is involved in anterograde and retrograde transport in axons via dynein and kinesin respectively. Pathologically paired helical filaments are formed, which are insoluble, self-assembled tau protein structures. Tau protein undergoes two post-translational modifications: hyperphosphorylation and truncation [16]. The hyperphosphorylation and abnormal tau aggregation, combined with its decreased clearance form NFTs and exert neurotoxicity in AD [21]. Hyperphosphorylation further halts microtubule binding, causing altered cytoskeleton stability and eventually loss of axon transport. Tau protein has been implicated in the disturbance of neuronal synchronization and hyperexcitability; along these lines, it could be connected to epilepsy [22]. Tau protein has also been embroiled in abnormal fiber growth and neuronal migration with hippocampal granule layer cell scatterings. These components are linked with epilepsy advancement [16].

Ion Channels

Beta-secretase 1 (BACE1) acts on Ion channels and primarily works on the β2 and β4 subunits of voltage-gated sodium channels [16]. BACE1 cleaves the β2 subunit regulating its transcription and expression on the cell surface; BACE1 also cleaves the channel β4 subunit, which mediates the closure of the voltage-gated sodium channel and, when cleaved, leads to aberrant firing and seizure-like activity. BACE1 levels are elevated in AD [16]. BACE1 also acts on voltage-gated potassium channels, which are linked to benign familial neonatal convulsions when mutated [16].

Gamma Amino Butyric Acid (GABA)

A few trials in AD patients and mice have shown that the collection of misfolded Aβ obstructs GABAergic interneuron action, causing impeded synaptic communication and loss of neural network activity, which ultimately leads to cognitive impairment [17]. A new report showed transcriptional downregulation of α1, α2, α3, α5, β1, β2, β3, δ, γ2, γ3and θ subunits of GABA A receptors and glutamate decarboxylase (GAD) chemical in the middle temporal gyrus (MTG) of post mortem brain samples from AD patients. These changes weaken the harmony among excitatory and inhibitory pathways that might cause cognitive impairment in AD [17]. Similarly, in biochemical examinations, the levels of GABA in the synapses were significantly lower in the CSF and the transient cortex of Alzheimer's patients, suggesting weakened synaptic action and neuronal transmission [17]. Soluble Aβ-actuated hyperexcitability has been related in vitro with a diminished GABAergic inhibition. This fortifies the hypothesis that GABA is one of the intermediaries associated with the organization and cell changes that lead to neuronal hyperexcitability in AD [16].

Glutamate

The capacity of L-glutamate (L-Glu) and various essential amino acids to excite CNS neurons was first exhibited in 1959. Since then, L-Glu has been distinguished as the core transmitter mediating fast excitatory synaptic reactions in the vertebrate central nervous system (CNS). L-Glu distribution inside the CNS is broad [18]. The neuronal cell loss in AD is mainly limited to cell bodies and dendrites of glutamatergic neurons in layers III and IV of the neocortex; loss of glutamatergically innervated cortical and hippocampal neurons are additionally noticed [18]. Disturbance of glutamatergic signaling towards a proepileptic state in AD has been connected to Aβ and Tau protein [16].

Role of Sleep

Interictal epileptiform discharges (IED) are significant intermittent electrophysiological events observed between seizures in patients with epilepsy. Based on a study by Vossel et al., the prevalence of IED detected with the help of routine scalp EEG in AD patients with seizures was about 30% [4]. Another study conducted by Diaz et al. has established that in epileptics and patients with IED, non-rapid eye movement (NREM) sleep is associated with a rise in focal and generalized epileptic discharges as opposed to awake state and rapid eye movement (REM) sleep [23]. This is further supported by other studies that have shown an increase in the epileptiform activity in AD patients with seizures during sleep EEG recordings [5,24]. But, it should also be noted that a normal EEG can be expected in 85% of epileptic patients with AD [25]. Therefore it can be concluded that the prevalence of epileptiform discharges is probably more than observed and also highlights the need for better biomarkers to detect seizures in AD. Further evidence suggests that most sleep-related subclinical epileptiform activity is present in many probable AD patients and recognition of the same would help improve diagnostics and patient outcomes [5,24].

Mutations

**Figure 2:**
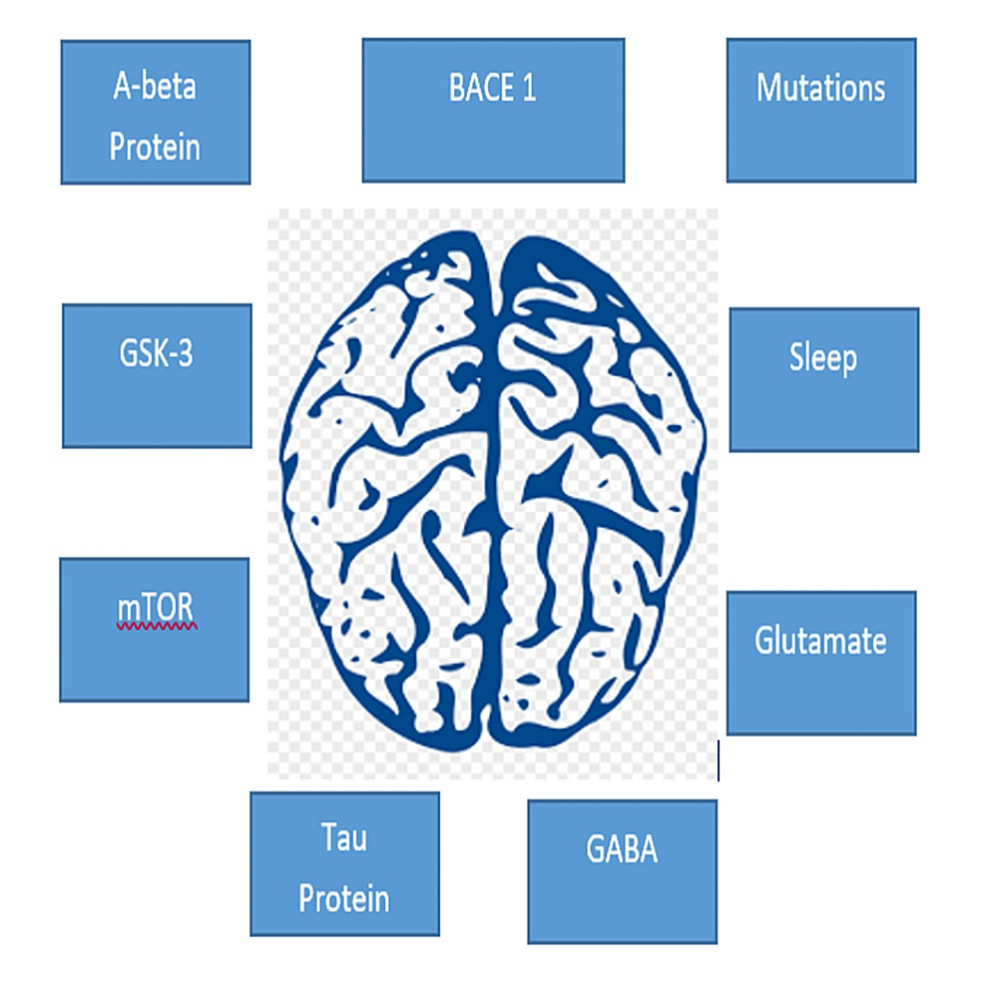
Pathogenesis of Alzheimer’s disease and epilepsy Figure by Manisha Purushotham BACE 1- Beta-secretase 1, GSK 3- Glycogen synthase kinase-3 , GABA- Gamma amino butyric acid, mTOR- Mammalian target of rapamycin

Fifty percent of patients with EOFAD have mutations either in presenilin 1 (PSEN1), presenilin 2 (PSEN 2), or amyloid precursor protein (APP) [26]. These mutations can cause increased amyloidogenic processing of APP and increased Aβ aggregates [27]. Mutations in PSEN1 are a significant cause of familial AD, as substantiated by 50% of the cases studied in nearly 480 families [27]. Figure 2 depicts the pathogenesis of AD and epilepsy in a concise way. 

Epidemiology

The prevalence of AD is inconsistent between several studies and ranges from 0.5% to 64% [28,29]. A meta-analysis published in 2021 found a combined seizure prevalence among patients with pathologically verified AD at 16% and in autosomal dominant AD (ADAD) at 2.8% to 41.7% [30]. It also established an incidence of 4.2 to 31.5 per 1000 person-years among those clinically diagnosed with AD [30]. Eleven percent of patients with adult-onset seizures had AD (95%CI, 7-14), with younger patients having an increased risk of seizures [30]. These differences in prevalence could be due to differences in diagnosing criteria, different databases used for studies, and variable disease severity [24]. It would also be challenging to obtain reliable history in more advanced stages of the disease. Conversely, symptoms such as syncope and behavioral changes can be falsely labeled as seizures. Hayashida et al. have described a case report of a 71-year-old AD patient who presented with symptoms of abdominal pain, nausea, vomiting, diarrhea, and bloating and was diagnosed to have abdominal epilepsy [31]. This further highlights the various ways epilepsy can manifest itself in AD, making the diagnosis challenging.

Risk factors

**Figure 3:**
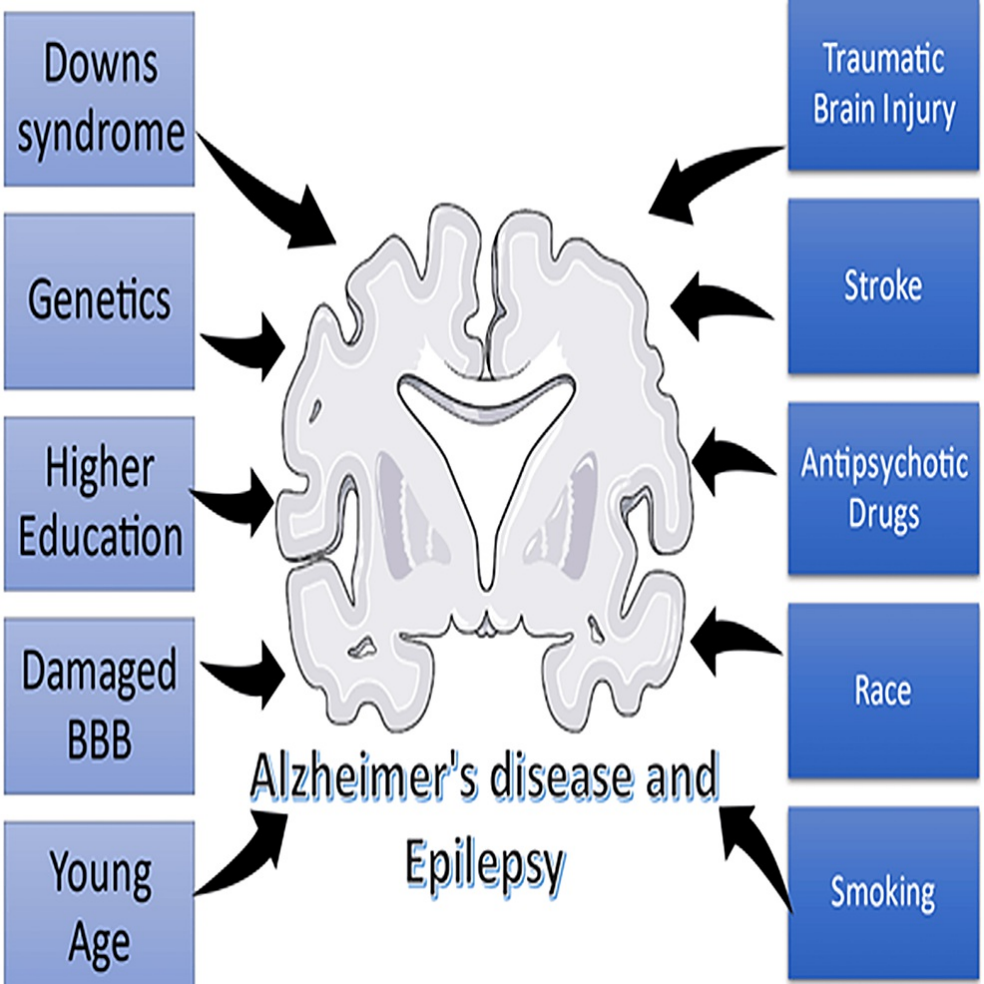
Risk factors for Alzheimer's disease and epilepsy Figure by Manisha Purushotham BBB- Blood brain barrier

There are common risk factors between AD and epilepsy as seen in Figure 3.

Identifying individuals with AD who are at an increased risk of developing seizures is pivotal in implementing strategies to slow down disease progression and improve outcomes. Filippov et al. proposed genotyping of the apolipoprotein E (APOE) allele in combination with EEG to identify early-onset AD [32]. This is further supported by a meta-analysis that showed that 15-45% of APOEε4 carriers had developed AD [33]. The incidences of AD are higher in APOEε4/4 patients (25-45%) who fall into the age group of 75-80 years compared with APOEε3/4 carriers (15-25%) when matched for age [34]. Recent studies in AD patients and animal models have established the role of blood-brain barrier (BBB) dysfunction in AD pathogenesis. Despite many studies, much remains to be found in our understanding of the BBB. These studies will be of immense value in detecting a potential risk factor for AD and as an important therapeutic modality. A study by Sweeney et al. suggests using in vitro human BBB models using pluripotent stem cells with neurodegenerative conditions to study this in greater detail [35]. Montagne et al. studied the permeability of the BBB using advanced dynamic contrast-enhanced magnetic resonance imaging (DCE-MI) showed age-dependent damage to the BBB in the hippocampus, the part responsible for memory, cognition, and learning [36]. Sleep disorders are also a risk factor for epilepsy as shown by a study conducted by Diaz et al. in which epilepsy was more prevalent in patients with insomnia or hypersomnia [23]. 

A 10-year cohort study by Lyou et al. showed that male sex, hypertension, hyperlipidemia, diabetes, and chronic kidney disease contributed to an increased risk of epilepsy [37]. It is crucial to note that this study was limited since it did not consider the influence of the environment, EEG findings, and the role of drugs. Literature shows that patients with Down syndrome (DS) have early onset of AD and seizures as the location of APP is on chromosome 21, and DS occurs due to a trisomy in chromosome 21 [38]. Antipsychotic drugs have been found to increase the risk of epileptic seizures. Second-generation antipsychotics, especially clozapine carry a higher risk than the first-generation antipsychotics [39]. Seizures occur in one to five of every 10 people who have had a traumatic brain injury (TBI), depending on the location of injury in the brain [40]. As noted before, age is a significant risk factor for AD [41]. Young age at the onset of AD, in both the sporadic and familial forms, was a risk factor and predictor for seizures and is perhaps one of the most consistent risk factors for AD [10]. A retrospective cohort study found that the black race, TBI, stroke, and pre-existing co-morbid depression were all critical risk factors for epilepsy in patients with AD [42]. This furthers the idea that the depression associated with AD could be due to degeneration of neurons and could point to a much more widespread insult to the brain, thus indicating a grimmer prognosis [43]. Smoking is said to be independently associated with both AD and epilepsy. In this case, epilepsy is believed to account for impaired neuron functioning and atherosclerotic changes in the brain's vasculature. However, a retrospective study found that these ischemic changes did not vary significantly among epileptic and non-epileptic subgroups [44]. Higher education is also believed to be a risk factor for seizures in AD, according to a study conducted by Horvath et al. [44].

Seizure semiology

AD can be classified as sporadic or familial and the semiology varies depending on the form of AD. The most common type of seizure in sporadic AD, accounting for about 55-70%, is the focal epileptic seizure that presents with an altered state of consciousness but with no motor component [4,10,41]. Other symptoms like déjà vu or jamais vu and staring episodes are also common and are wrongly interpreted as cognitive fluctuations [4]. In familial ADAD, motor symptoms are more frequently seen as focal or tonic-clonic, or myoclonic seizures [30,42,43].

Diagnosis

Diagnostic challenges include identifying the best biomarkers for measuring sub-clinical epileptiform discharges and finding modalities for early detection.

Myelin Sheath Imaging

In their study, Drenthen et al. stated that reduced myelin content is commonly associated with epilepsy and other neurodegenerative conditions such as AD. Myelin sheath studies include imaging methods such as magnetic resonance imaging (MRI) and positron emission tomography (PET) scans and histopathological methods such as immunohistochemistry and Western blot. Thus, making it a potential candidate for a proper diagnostic procedure [45].

Scalp EEG

Many studies show that epileptiform activity is often undiagnosed in AD, and here EEGs are of great value especially when taken overnight during sleep. In a study, Horvath et al. aimed to identify subclinical epileptiform activity (SEA) in patients with AD and its effect on the progression of the disease. They examined 52 Alzheimer patients and 20 healthy individuals. They found that SEA changes are associated more significantly and frequently in patients with AD and are accompanied by decreased cognitive ability and memory impairment. Thus, SEA may not present clinically and is present in only 50% of patients with AD, but its association with rapid deterioration of the disease indicates the importance of its early detection by EEG [46]. When monitored for a minimum duration of eight hours or by including sleep, the sensitivity was improved to 60-80% [12,24]. Serial EEGs are also helpful in diagnosis as opposed to standard 30 minutes EEG [12]. A study by Lam et al. included 24-hour ambulatory EEG monitoring of patients with AD and healthy elderly individuals. This study aimed to find how some EEG findings correlate to the clinical presentations of seizures in AD and concluded that left temporal hyperexcitability on EEG was seen in the early stages of AD, with clinical seizures more commonly associated with bitemporal lobe excitability [47]. 

CSF Biomarkers

A study conducted by Cretin et al. evaluated CSF findings of epileptic and nonepileptic Alzheimer patients and found no significant differences in the levels of neurodegenerative biomarkers and albumin. They also found a significant correlation between the presence of CSF amyloid proteins and epileptic patterns on EEG. However, the authors concluded that CSF analysis could not serve as a substitute biomarker since there can be other causative factors apart from an amyloid-centric pathology, thus making this diagnostic modality inferior to others [25].

Functional Magnetic Resonance Imaging (fMRI)

According to Dickerson et al., fMRI can identify pathologies of AD and epilepsy in frontal, temporal, and parietal cortices and can also identify areas in the brain showing hypoactivation due to atrophy and compensatory hyperactivation. It helps identify large-scale functional abnormalities and minute neuronal dysfunction in the early stages of AD [48].

*Magnetoencephalogram* *(MEG)*

Kitchigina et al., in their study, stated that MEG could be very useful in the early detection of temporal lobe epilepsy and AD since it has the potential to detect changes occurring in the early stages of the disease. MEG can also see alterations in theta and gamma rhythms familiar to AD and epilepsy [49].

Foramen Ovale Electrodes

Lam et al., in their study, used foramen ovale electrodes to study electrical changes in two patients with AD and found hippocampal seizure activity and epileptiform spikes, which did not have any clinical presentation. Thus, this is a helpful modality in detecting occult hippocampal activity in the early stage of the disease [50].

Genetic Studies

Juzwik et al. stated that microRNAs are common genetic markers that are dysregulated in various neurodegenerative disorders, including AD and epilepsy, and thus can be used as a potential biomarker for their diagnosis [51].

PET Scan

A review by Cai et al. talks about the role of PET imaging in the detection of synaptic vesicle glycoprotein 2A (SV2A) to study many neuropsychiatric conditions, one of which is AD. It could potentially be a reliable biomarker due to synaptic abnormalities in all neuropsychiatric disorders [52]. 18F-labeled fluoro-2-deoxyglucose positron emission tomography (FDG-PET) is also believed to help localize epileptogenic foci, especially in patients with underlying AD or other neurodegenerative diseases, especially if surgical treatment is warranted [53]. Altered adenosine receptor expression, a feature of many neurological conditions, including AD, can also be detected using a PET scan [54].

Cognitive Testing

Cretin et al., in their study, stated that epilepsy occurring in sporadic AD could be associated with other symptoms such as amnesia and behavioral changes. This can be a factor that can lead to a misdiagnosis if clinical cognitive assessment is used as the only diagnostic modality. Moreover, epilepsy is seen in various stages of sporadic AD, mild, moderate, and severe, thus rendering it nonspecific and necessitating other modalities such as brain imaging [55]. The diagnostic modalities are summarized in Table 2.

**Table 2:** Summary of important points from various diagnostic modalities PSWE: Paroxysmal slow wave events, EEG: Electroencephalogram, MEG: Magnetoencephalogram, AD: Alzheimer's disease, BBB: Blood Brain Barrier, MCI: Mild cognitive impairment

DIAGNOSTIC MODALITIES	IMPORTANT POINTS
Scalp EEG	More diagnostic when used during sleep and overnight. Subclinical epileptiform activity associated with cognitive decline can be detected. PSWEs indicative of BBB dysfunction can be seen. EEG changes of bitemporal lobe excitability were associated with clinical seizures [46,47].
Functional Magnetic Resonance Imaging	Has shown to increase hippocampal activation that predicted mesial temporal lobe failure and cognitive decline at the MCI stage of AD. Can detect hypoactive areas due to atrophy and compensatory hyperactive areas as well [48].
Magnetoencephalogram (MEG)	A study showed that using one hour MEG and overnight scalp EEG in early onset AD found epileptiform discharges in 21% of participants while none was visible on scalp EEG. Potential to detect early changes that occur during the disease process [49].
Foramen Ovale Electrodes	Gold standard for assessing deep temporal epileptiform activity. A study showed that patients with AD showed mesial temporal lobe seizures that were not seen on scalp EEG . Can also detect occult hippocampal activity in early stages [50].
Positron Emission Tomography Scan (PET Scan)	18F-fluorodeoxyglucose (FDG) PET Is helpful in both dementia and epilepsy but has not yet been evaluated in AD-related epilepsy. Can detect altered synaptic vesicle glycoprotein 2A and altered adenosine receptor expression, derangements of both are seen in various neurological disorders [52-54].

Management

Managing epilepsy in patients with AD can be especially challenging as the disease itself is a risk factor for epilepsy [56] and the concerned population is more prone to drug interactions due to different pharmacokinetics and effects on the CNS [57,58]. Therefore, the objective of antiepileptic drugs (AEDs) treatment in this group takes into account several factors beyond adequate seizure control [58]. Patients without witnessed seizures but positive epileptiform activity should be treated based on clinician judgment for example if there is concern that it is causing impairment of cognition [4]. Slow titration and monotherapy with vigilant monitoring of side effects are general recommendations [56].

Consideration of AD Drugs in Seizures

It is important to consider that some of the medications for Alzheimer’s can lower the seizure threshold. While one study reports that memantine can lower seizure threshold [59], another study reports statistically significant benefits of using memantine to improve cognition in epileptic patients with a good safety profile and no noted adverse effects [60]. Out of all the adverse drug reactions noted on donepezil and rivastigmine, 8.4% and 6.4% respectively were accounted for by convulsions [61]. On the other hand, no increased frequency of seizures was noted in the randomized, double-blind, placebo-controlled study of donepezil [62]. Galantamine was deemed to be safe in epilepsy and aducanumab was approved in 2021 for AD but needs further research on this topic [63,64]. Typical antipsychotics and bupropion commonly used for depression and mood stabilization can also lower the seizure threshold in these patients [65]. AEDs remain the intervention of choice with an excellent response rate of 79%, defined as a 95% reduction in seizures or fewer than three seizures annually [56,66]. Clinical judgment should be exercised for individual patients. No Empirical treatment with AED is indicated in patients without clinical or electrographic signs of network hyperexcitability but can be started if concerned that it is causing cognitive impairment [59]. In symptomatic AD patients, the risk of recurrence after the first unprovoked seizure stands at 70% and it is recommended to treat them indefinitely [67].

Pharmacological Interventions

Levetiracetam: A comparatively well-studied AED with proven efficacy and a good safety profile in AD. It can be used for both generalized and focal seizures and has fewer drug interactions [56]. One parallel randomised controlled trial (RCT) comparing lamotrigine (LTG), levetirecetam (LEV), and phenobarbital (PB) reported that around 71% (27 out of 38 people) of patients were responders on LEV monotherapy, with doses ranging from 500 mg to 2000 mg followed for a year after a month of dose adjustment [68]. Another prospective open-label study investigated 25 patients with advanced AD and noted a similar response rate of 72% with doses of 1000-1500mg and followed the patient for a year after giving a four week period for adjustment, while 16% discontinued due to issues with tolerability [69]. LEV was also noted to have an improvement in cognition, spatial memory, and executive function in AD with epileptiform activity. It also showed improved scores on mini-mental state examination (MMSE) and Alzheimer's Disease Assessment Scale-Cognitive Subscale (ADAS-cog) [59,69,70]. The medication was also well tolerated with no participant discontinuing treatment due to adverse effect [13]. Low doses of 125 mg twice a day was shown to improve hippocampal-based memory performance [13]. Levetiracetam also does not affect cytochrome p450 function and is exclusively renally excreted, hence accounting for fewer drug interactions [71] though, it is well established that LEV is noted for behavioral side effects, including irritation, agitation and even depression [72].

Lamotrigine: Another excellent option with a good safety profile and efficacy similar to LEV is lamotrigine with a response rate of 59% in a parallel RCT by Cumbo et al. [12,57]. It was also shown to improve cognition, mood, and performance on recognition and naming tasks as seen in a study by Tekin et al. after eight weeks of a trial of 300 mg of lamotrigine [73].

Gabapentin (GBP): Rowan et al. noted that 1500 mg per day of gabapentin had comparable efficacy with LTG 150 mg per day in older adults with a mean age of 72 years with mild cognitive impairment in a double-blinded RCT with 593 participants. While this is not specific to AD, it is a potential option [74]. Participants were followed for 12 months and remained seizure-free. GBP use in advanced AD remains questionable. Notable side effects include drowsiness and weight gain [75]. Hommet et al. reported similar findings though tolerance was better than LTG [75].

Carbamazepine (CBZ): Double-blinded RCT showed comparable efficacy of LTG, GBP, and carbamazepine at 600 mg per day but the tolerability was lowest [75]. CBZ, also being an enzyme inducer, was noted to be associated with osteomalacia, hence vitamin D, calcium, and physical therapy should be co-administered. Other side effects include cardiac dysfunction and low sodium [59].

Phenobarbital: It was seen to have an effective response of around 64 % similar to lamotrigine and levetiracetam in a randomized case-control study with a dose of 50-100 mg per day followed for a 12-month period. The major side effect was somnolence in nearly one-third of the patients. Cognitive outcome was poorer when compared to levetiracetam and lamotrigine. Studies suggest against its use for the abovementioned side effects but also due to increased risk of osteomalacia, ataxia leading to greater risk of fractures [59].

Valproate: It was associated with significant side effects including increased cortical volume loss, worsened MMSE and cognition, tremor, and gait disturbance compared to placebo in patients with AD which provide cases for not using it as your drug of choice [76,77].

Phenytoin: The efficacy of phenytoin remains controversial and variable with a lack of RCTs, and significant side effects including cognitive decline, ataxia, and sedation which are severe concerns in existing Alzheimer's [67,72]. Phenytoin was seen to increase seizure risk in mouse models [78].

Benzodiazepines: While highly efficacious in terminating seizures, they are well known to cause cognitive decline, delirium, and withdrawal seizures [79,80]. They also have significant addictive potential and hence should be only used as a last resort. Interestingly, long-term use has also been linked to the development of AD with one study reporting an odd’s ratio of 1.51 [81]. Other AEDs including oxcarbazepine and lacosamide have been used to treat seizures, but insufficient data is available for their efficacy in Alzheimer’s.

Levetiracetam (LEV) and lamotrigine have currently been shown to be the most effective treatment for seizures in patients with AD with levetiracetam also showing positive effects on cognition and lamotrigine helps stabilize mood [57,61,72,73]. Phenytoin and phenobarbital are not recommended for epilepsy in AD due to negative cognitive effects. The response to treatment is, however, good as can be seen summarized in Table 3.

**Table 3:** Summary of randomized control trials including the various drugs used to treat epilepsy

Study	Year	Design	Population	Conclusion
Vossel et al. [70]	2021	Randomized control trial.	34 adults out of which 13 had epileptiform changes. Only 28 completed the study out of which 10 had epileptiform changes during enrolment into the study.	Levetirecetam was found to have a positive effect on spatial memory, executive functions and epileptiform activity in patients with AD.
Vossel et al. [12]	2013	Retrospective observational study	54	Good seizure control and tolerability with lamotrigine and levetiracetam.
Cumbo and Ligori. [68]	2010	Prospective randomized three arm parallel group study	95, randomly assigned to a one of LEV(levetirecetam), PB(phenobarbital) or LTG(lamotrigine).	All 3 medications were equally effective. LEV- fewer adverse effects, improved cognition,improved attention. LTG- positive effect on mood PB-negative cognitive effects
Rao et al. [66]	2009	Retrospective study	39	Several antiseizure medications were used and 79% of the participants. had an excellent response to AED therapy
Belcastro et al. [69]	2007	Prospective observational study	25	LEV monotherapy was given and 72% of patients were seizure-free for at least one year

Non-pharmacological Interventions

Non-pharmacological interventions, such as transcranial magnetic stimulation (TMS), deep brain stimulation, chiropractic care, Reiki, and acupuncture, have been shown to improve outcomes in people with AD or epilepsy [54-56]. Stem cell therapy has shown independent benefit in cases of both epilepsy and AD in rodent model studies and thus could potentially be explored as a future modality [82].

Limitations

AD and epilepsy have a complex association and although a lot of studies have established an association, the mechanisms underlying the same are still not fully understood. In this review, we have discussed the existing literature with respect to epidemiology, risk factors, possible mechanisms, diagnosis, and treatment. We have only briefly discussed the latest clinical trials without going into the details of the mechanisms of individual AEDs. We have also not discussed upcoming treatment options such as anti-amyloid treatment, GSK3 inhibitors, and Tau inhibitors due to insufficient evidence on their efficacies. At present, it is difficult to give recommendations on treatment strategies in AD patients with epilepsy as there are insufficient trials and data is still evolving.

## Conclusions

The studies reviewed in this article clearly show an association between AD and epilepsy and more so with the early onset familial autosomal dominant form of AD. It is now known that seizures can precede neuronal loss. Most seizures are subclinical making diagnosis a challenge as they may present solely as cognitive fluctuations which can be overlooked in the background of AD. The risk factors were then discussed as there are some modifiable risk factors that may help slow the disease progression. More trials are needed to substantiate the same and to prove clinical significance. We also discussed some diagnostic modalities that help in detecting seizures earlier so that AEDs can be initiated to halt disease progression by possibly affecting network hyperexcitability and improving outcomes. Currently, levetiracetam and lamotrigine have the best evidence for use in AD patients with seizures. In-depth research is required to better understand the mechanisms that associate AD with epilepsy and more clinical trials on AEDs are required to devise a standardized treatment plan for seizures in AD.
